# Suicide deaths associated with climate change-induced heat anomalies in Australia: a time series regression analysis

**DOI:** 10.1136/bmjment-2024-301131

**Published:** 2024-08-09

**Authors:** Lucas Hertzog, Fiona Charlson, Petra Tschakert, Geoffrey G Morgan, Richard Norman, Gavin Pereira, Ivan C Hanigan

**Affiliations:** 1 School of Population Health, Curtin University, Perth, Western Australia, Australia; 2 WHO Collaborating Centre for Climate Change and Health Impact Assessment, Perth, Western Australia, Australia; 3 Healthy Environments and Lives (HEAL) National Research Network, Perth, Western Australia, Australia; 4 Queensland Centre of Mental Health Research and School of Public Health, The University of Queensland, Brisbane, Queensland, Australia; 5 School of Media, Creative Arts and Social Inquiry, Curtin University, Perth, Western Australia, Australia; 6 School of Public Health, Faculty of Medicine and Health, University of Sydney, Camperdown, New South Wales, Australia; 7 University Centre for Rural Health, Faculty of Medicine and Health, University of Sydney, Lismore, New South Wales, Australia; 8 Centre for Safe Air, NHMRC CRE, Sydney, New South Wales, Australia; 9 School of Population Health, Faculty of Health Sciences, Curtin University, Perth, Western Australia, Australia; 10 enAble Institute, Faculty of Health Sciences, Curtin University, Perth, Western Australia, Australia

**Keywords:** suicide & self-harm, data interpretation, statistical

## Abstract

**Background:**

Although environmental determinants play an important role in suicide mortality, the quantitative influence of climate change-induced heat anomalies on suicide deaths remains relatively underexamined.

**Objective:**

The objective is to quantify the impact of climate change-induced heat anomalies on suicide deaths in Australia from 2000 to 2019.

**Methods:**

A time series regression analysis using a generalised additive model was employed to explore the potentially non-linear relationship between temperature anomalies and suicide, incorporating structural variables such as sex, age, season and geographic region. Suicide deaths data were obtained from the Australian National Mortality Database, and gridded climate data of gridded surface temperatures were sourced from the Australian Gridded Climate Dataset.

**Findings:**

Heat anomalies in the study period were between 0.02°C and 2.2°C hotter than the historical period due to climate change. Our analysis revealed that approximately 0.5% (264 suicides, 95% CI 257 to 271) of the total 50 733 suicides within the study period were attributable to climate change-induced heat anomalies. Death counts associated with heat anomalies were statistically significant (p value 0.03) among men aged 55+ years old. Seasonality was a significant factor, with increased deaths during spring and summer. The relationship between high heat anomalies and suicide deaths varied across different demographic segments.

**Conclusions and implications:**

This study highlights the measurable impact of climate change-induced heat anomalies on suicide deaths in Australia, emphasising the need for increased climate change mitigation and adaptation strategies in public health planning and suicide prevention efforts focusing on older adult men. The findings underscore the importance of considering environmental factors in addition to individual-level factors in understanding and reducing suicide mortality.

**WHAT IS ALREADY KNOWN ON THIS TOPIC**

- Environmental factors influence mental health and suicidality, but the specific impact of climate-related heat anomalies on suicide rates has been less explored.

**WHAT THIS STUDY ADDS**

- Our study estimates that 0.5% of suicides in Australia from 2000 to 2019 were attributable to climate change-induced heat anomalies, with a notable impact among older adult men.

**HOW THIS STUDY MIGHT AFFECT RESEARCH, PRACTICE OR POLICY**

- The study findings emphasise the need for public health strategies that integrate climate adaptation measures, particularly focusing on vulnerable populations during peak heat periods to reduce suicide risks.

## Background

 The escalating impacts of global climate change on health and well-being are gaining attention, underscoring the need for a deeper understanding of its effects on individual susceptibility to a range of mental health outcomes and suicidality.^1^,^4^ Recent decades show a global decrease in suicide rates, an encouraging sign amid persistent public health challenges.^5^ This positive shift, however, does not lessen the complexity of factors contributing to suicide risk, including environmental factors that are often mentioned in studies but seldom thoroughly investigated.^6^ Climate change-induced high heat anomalies are an environmental hazard that has been intensifying over recent decades with global warming.

Suicide is a multifaceted phenomenon influenced by social, economic, psychological and environmental factors. While traditional research has often focused on clinical and psychological aspects,^6^ the environmental determinants, particularly those related to climatic hazards, have remained relatively underexamined. This oversight is significant, considering the growing evidence suggesting a link between temperature extremes, exacerbated by climate change and suicide.^7^ The interplay between rising temperatures and increased well-being risks is a critical area for investigation, especially in light of the broader impacts of climate change on natural and human systems.

Emerging research has begun to shed light on the relationship between temperature fluctuations and suicide rates, revealing a complex and nuanced interaction. Studies from various geographical settings suggest that both extreme heat and significant deviations from average temperature conditions can influence the incidence of suicide.^8 9^ These findings point to the potential for temperature anomalies to exacerbate individual vulnerabilities and increase the risk of suicidal behaviour.

This study aimed to explore the relationship between Australian climate change-induced heat anomalies and suicide across greater capital city statistical areas (GCCSAs) and non-metropolitan areas (rest of state regions). We examined the association between suicide and anomalous heat conditions (compared with the historical period since 1950–2019). Our research aims to contribute to the growing body of knowledge on the impacts of observed climate change on health and well-being.^10 11^

## Methods

### Population data and study region

Our population-based study encompasses GCCSAs from states and territories of australia, namely Sydney and the rest of New South Wales, Melbourne and the rest of Victoria, Brisbane and the rest of Queensland, Adelaide and the rest of South Australia, Perth and the rest of Western Australia, Hobart and the rest of Tasmania, Darwin and the rest of the Northern Territory and the Australian Capital Territory, excluding external offshore territories. Population data was retrieved from the Australian Bureau of Statistics at the State Area Level geographic boundary system from the 2016 Census (a representative census for the period).^12^

### Suicide data

Data on monthly counts of deaths by State were obtained from 2000 to 2019 from the Australian Clean Air Research Data Analysis Technology platform hosted dataset provided by the Australian Institute of Health and Welfare.^13^ Using codes from the International Statistical Classification of Diseases and Related Health Problems 10th Revision, we selected causes of death attributed as intentional self-harm (X60–X84) and sequelae of intentional self-harm (Y87.0), disaggregated by sex (male and female) and age groups (0–29, 30–54 and 55+ years).

### Temperature data and maximum temperature anomaly estimation

Gridded climate data of site-based surface observations or satellite data from the Australian Gridded Climate Data were obtained for each pixel of a grid with a resolution of 5°×5° (approximately 5×5 km) using a spatial model for Australia.^14^ We estimated monthly minimum and maximum average temperature in degree Celsius by GCCSA. The exposure of interest (maximum temperature anomaly) was estimated by calculating the difference between the average maximum temperature for each specific month and GCCSA and the long-term monthly average maximum temperature from available data from 1950 to 2019. This approach allowed to quantify deviations of maximum temperatures from their historical averages. This enabled the identification of periods of above-average heat measured in degree Celsius. The anomalies were computed for each month and GCCSA to provide a detailed spatiotemporal characterisation of temperature variations across Australia over the study period. Positive values of the anomaly indicated temperatures higher than the historical monthly average, serving as a key metric in evaluating the extent and intensity of heat events. Negative temperature anomalies were excluded from this analysis, as we focused on capturing the relationship between heat and suicide.

### Statistical analyses

We used a generalised additive model (GAM) to fit penalised regression splines in a time series regression to explore the relationship between heat anomalies and suicide, accommodating seasonality and trend and potential non-linear relationships with heat across different demographic groups.^15 16^ This approach has been employed in previous research focusing on environmental stressors, such as drought, and their link to suicide^2^ and mental health outcomes at the population level.^3^ We assessed the association of suicide counts with heat anomalies using a Poisson distribution within the GAM framework, suitable for the rare-event discrete count suicide data type. Diagnostics were conducted to ensure the appropriateness of this model, including assessment of overdispersion often associated with rare disease count data and the application of the quasi-Akaike Information Criterion (QAIC) to evaluate model fit and complexity, enabling a more accurate comparison of models with and without the inclusion of the interaction terms. Our analysis incorporated temperature anomalies as experienced by males and females across three age groups: 0–29, 30–54 and 55 years and above, by GCCSA.

Within the GAM, predictors were modelled using cubic splines with 3 df to explore potential non-linearity. Cubic splines were used to balance smoothness and flexibility in fitting the data.^17^ Furthermore, we integrated a natural spline for the year variable to account for long-term temporal trends, again with 3 df. An offset term was included to account for population size variations across observations, allowing the model to adjust the estimated suicide rates relative to the population size, ensuring comparability across different geographic areas and population densities. This design was further enhanced by introducing interaction effects between age group, sex, and year and including a fixed effect for the GCCSA. Seasonal effects were captured using a cyclic cubic regression spline for the month, ensuring a fluid transition between the end and beginning of the year and accommodating the cyclical nature of seasonal trends in suicides.^16^

### Sensitivity analysis

To assess the effect of temperature anomalies on the number of suicides, we contrasted actual and counterfactual scenarios. The actual scenario used the observed data, including temperature anomalies. In contrast, we set temperature anomalies for the counterfactual scenario to zero across all GCCSAs. This methodological design aimed to simulate an environment without the influence of high heat climate change-induced temperature anomalies, serving as a baseline for comparison.

Predictions of suicide counts were generated for both scenarios using the GAM, facilitating an evaluation of the impact of climate change temperature anomalies. The attributable number of suicides was determined by calculating the difference between the predicted counts under actual conditions, with temperature anomalies and those under the counterfactual scenario, which assumes no heat anomalies. This comparative analysis allowed quantification of the specific contribution of temperature anomalies to the overall number of suicides, providing a clearer understanding of the relationship between heat anomaly and suicides.

A bootstrapping procedure was employed to quantify the uncertainty associated with our estimates. This involved resampling the dataset 1000 times with replacement and recalculating attributable numbers for each sample. From the bootstrapped data, a 95% CI was derived, capturing the 2.5th and 97.5th percentiles and delineating the range within which the actual value of attributable suicides likely falls. All analyses were conducted in R, using the packages *mgcv* and *splines*.

## Results

### Descriptive statistics

Table 1 presents the population size, the total number of suicides and the suicide rates disaggregated by sex for Australian GCCSAs from 2000 to 2019. Overall, the data encompass a total population of 23 351 004 individuals, with 11 833 663 females and 11 517 329 males. During this period, there were 50 733 recorded suicides in total, with 11 932 (23.5%) female and 38 802 (76.5%) male suicides.

**Table 1:** Population, suicide deaths and suicide death rates by Australian states and territories, 2000 to 2019

GCCSA	Population			Suicide deaths			Suicide deaths rate*		
	Female	Male	Persons	Female	Male	Persons	Female	Male	Persons
Sydney	2 447 221	2 376 766	4 823 991	2069	5997	8066	4.23	12.61	8.36
Rest of NSW	1 341 813	1 301 717	2 643 536	1259	4971	6229	4.69	19.09	11.78
Melbourne	2 285 616	2 199 597	4 485 211	2101	5764	7865	4.60	13.10	8.77
Rest of VIC	729 694	704 128	1 433 818	697	2759	3456	4.77	19.59	12.05
Brisbane	1 153 351	1 117 452	2 270 800	1175	3872	5048	5.09	17.33	11.11
Rest of QLD	1 222 294	1 197 426	2 419 724	1501	5316	6817	6.14	22.20	14.09
Adelaide	661 823	633 888	1 295 714	754	2342	3096	5.70	18.47	11.95
Rest of SA	187 706	190 368	378 074	183	799	982	4.89	20.98	12.99
Perth	980 326	963 536	1 943 858	1140	3320	4460	5.81	17.23	11.47
Rest of WA	253 230	270 936	524 167	341	1278	1619	6.74	23.58	15.44
Hobart	114 422	107 930	222 356	183	417	600	8.00	19.32	13.49
Rest of TAS	145 646	140 977	286 627	179	697	876	6.14	24.72	15.28
Darwin	64 939	71 889	136 828	82	345	426	6.30	23.97	15.58
Rest of NT	44 157	45 287	89 443	95	366	461	10.76	40.41	25.77
Canberra	201 425	195 432	396 857	173	559	732	4.29	14.30	9.22
Australia†	11 833 663	11 517 329	23 351 004	11 932	38 802	50 733	5.88	20.46	13.16

According to the Census 2016 methodology, if sex was not stated it was imputed.^39^

* Average rates per 100 000 for the study period.

† The total number of persons might differ from male plus female, as sex was not stated in some cases.

GCCSA, greater capital city statistical area; NSW, New South Wales; NT, Northern Territory; QLD, Queensland; SA, South Australia; TAS, Tasmania; VIC, Victoria; WA, Western Australia.

Suicide rates per 100 000 population showed considerable regional variation. The highest male suicide rate was observed in the rest of Northern Territory (40.41), which may reflect high suicide rates among the indigenous populations in the area.^18^ For females, the highest rate was also found in the rest of Northern Territory (10.76), surpassing the national average of 5.88 for females. This results in the rest of Northern Territory having the highest combined suicide rate of 25.77. Conversely, the lowest suicide rates for both males and females were recorded in Sydney, with 12.61 for males and 4.23 for females per 100 000 population.

At the national level, the average suicide rate for males was significantly higher than females, with rates of 20.46 and 5.88, respectively, resulting in a combined average suicide rate of 13.16 per 100 000 population over the study period.

**Figure 1:**
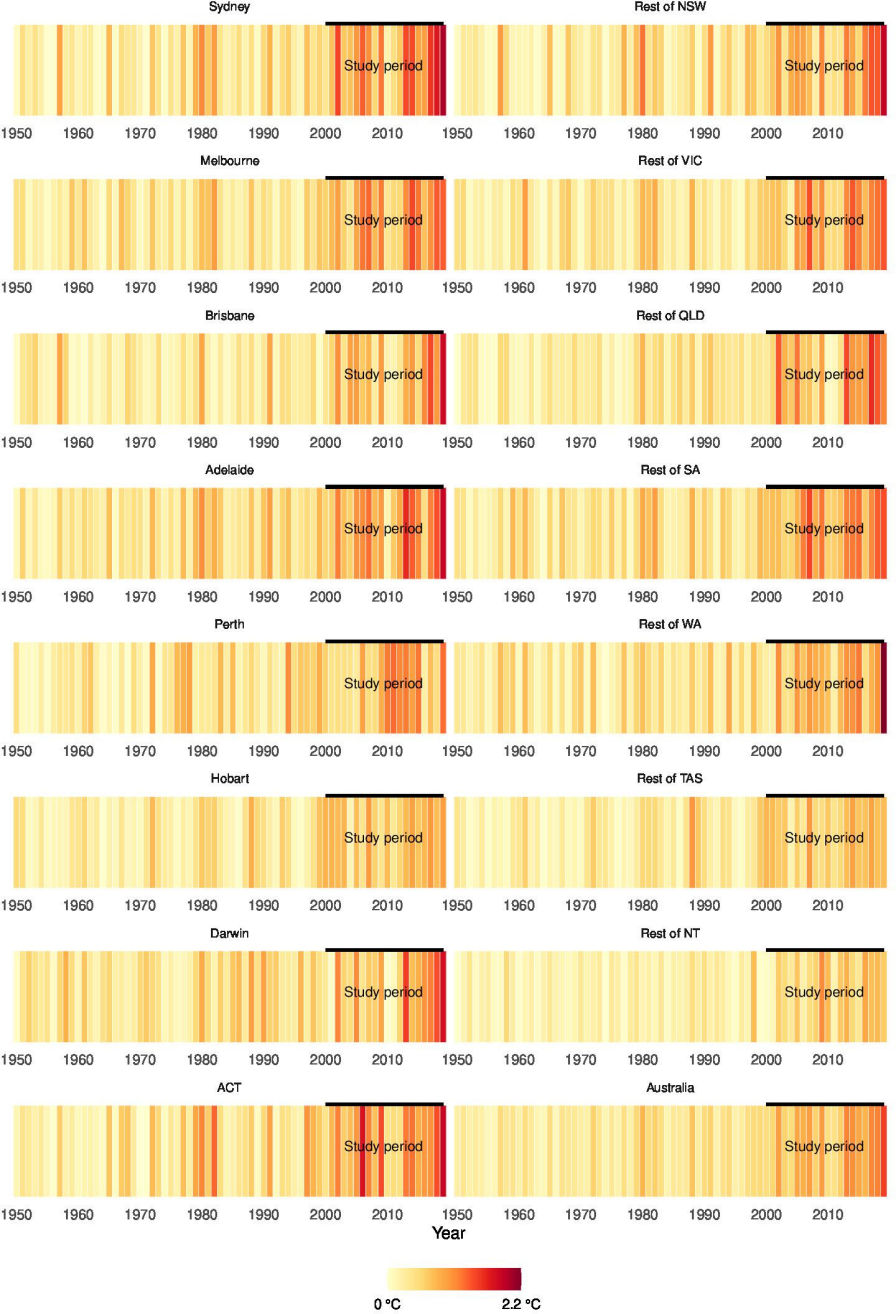
Positive yearly average maximum temperature anomalies in Australia (compared with the historical maximum averages in the selected period, 1950–2019)*. *Yearly averages are for illustration and context, as calculations used monthly data. NSW, New South Wales; NT, Northern Territory; QLD, Queensland; SA, South Australia; TAS, Tasmania; VIC, Victoria; WA, Western Australia.

Considering all regions, high heat anomalies in the study period were between 0.48°C and 1.48°C hotter than the historical period from 1950. Figure 1 illustrates the trend of positive yearly average maximum temperature anomalies in Australia by GCCSA over 70 years from 1950 to 2019, highlighting the study period from 2000 to 2019 most impacted by climate change. The figure is a visual representation of the deviation from historical maximum temperature averages, categorised by colour intensity to represent the magnitude of the anomaly. This warming trend becomes more pronounced in the years leading up to and including the study period, suggesting a substantial increase in temperature anomalies during these years.

### Relationship between monthly number of suicides and anomalous heat

**Figure 2:**
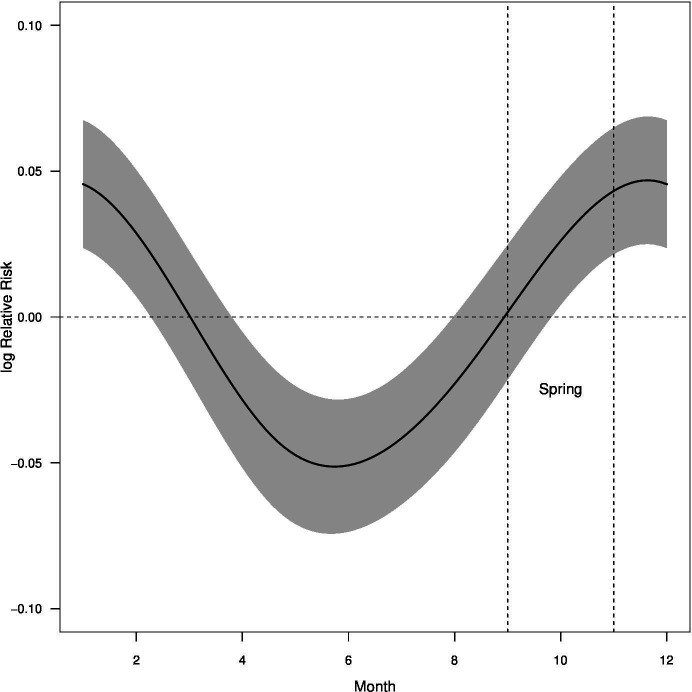
Monthly variation of log relative risk of suicide in Australia with peaks in spring and early summer.

The GAM multivariate analysis for the whole country shows an increase in relative risk for suicide associated with seasonality, with heightened risks during spring and summer (figure 2). The model incorporates the effect of smoothed maximum temperature anomaly and interactions between age group, sex, year and GCCSA. The Pearson χ^2^ statistic for overdispersion, conducted to ensure the appropriateness of this model, resulted in a value of 1.08, indicating that our data do not exhibit significant overdispersion and substantiating our use of the Poisson family in the model.^19^ Additionally, the ΔQAIC between our base and interaction-augmented models was 1485.4, significantly favouring the latter. This underscores the enhanced model’s better fit and justifies incorporating age, sex and regional interactions. The cyclic cubic spline was employed to model the cyclical effect of the month, effectively adjusting for the seasonality in suicide risk.

**Figure 3:**
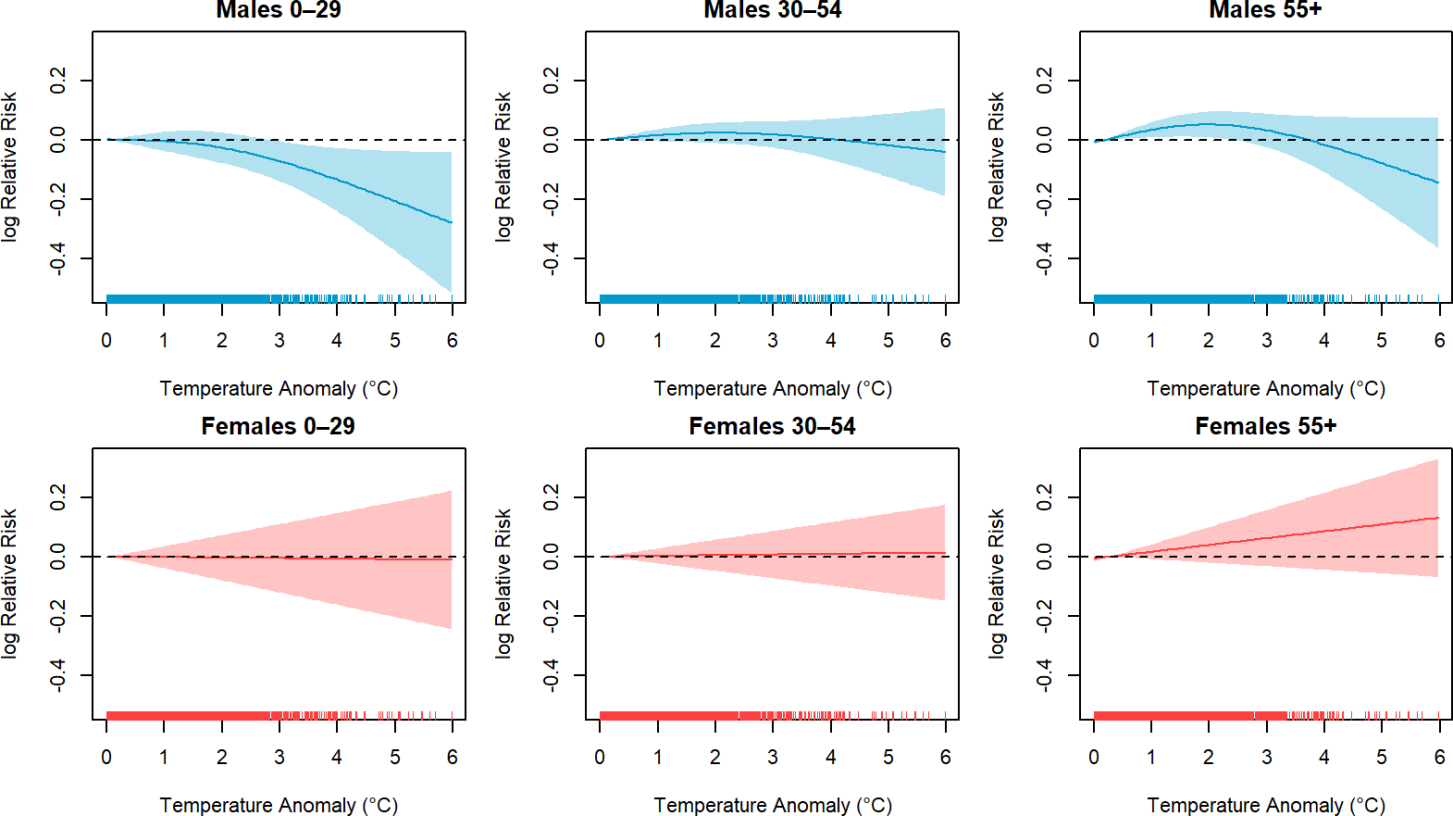
Association between suicide risk and heat anomaly disaggregated by sex and age groups across Australia. Shaded areas represent 95% CIs. Tick marks on the x-axis represent the distribution of data points.

To estimate the association of suicide with climate change-induced high-temperature anomalies, the GAM model was stratified by sex and three distinct age groups. The analysis accounts for a smooth function of heat anomalies and their interactions with sex and age groups, while adjusting for seasonality and regional differences. Given the use of non-linear semiparametric splines in the model, presenting the association as relative risks poses statistical challenges. Therefore, in figure 3, we visualise the estimated exposure–response functions with their corresponding CIs, disaggregated by age groups.

Figure 3 shows non-linear associations with increased relative risk of suicide for heat anomalies between 0°C and 3°C in the 30–54 and 55+ age groups for men, with a subsequent plateau beyond this range. The exposure–response function for women aged 55+ years also implied increasing relative risks with increases in heat anomaly, but this was not statistically significant at the p<0.05 threshold. However, for males aged 0–29, the association was inverse, demonstrating a decrease in relative risk with increasing heat anomalies above the threshold of 3°C, although it is noteworthy that there were fewer data points observed above this level of exposure. Age stratification by sex and GCCSA region is shown in online supplemental file 1, with results that can be assessed by combining age, sex and region-specific analysis.

### Attributable number of suicides due to anomalous climate change-induced heat

Table 2 presents the number of suicide deaths attributable to heat anomalies in the study period and provides a complementary view of the non-linear effect estimates shown above. Our model reveals age groups and sex differences in these numbers. The estimates are presented along with 95% CIs estimated using a bootstrapping procedure. The estimates indicate 264 male suicide deaths attributable to heat (95% CI 257 to 271) in the age groups 55+ years, with a significant p value of 0.03 considering statistical significance as p<0.05.

**Table 2:** Estimated suicide deaths attributable to temperature heat anomalies in Australia, 2000–2019

Age group, years	Suicide deaths (95% CI)			
	Male	P value*	Female	P value
0–29	−117 (−128 to −106)	0.0559	−5 (−5 to −4)	0.936
30–54	198 (189 to 208)	0.3156	12 (12 to 13)	0.8751
55+	**264 (257 to 271)**	0.0325*	71 (69 to 73)	0.1906

Bold values indicate significance at p<0.05.

* P<0.05.

The model also suggests other trends, despite not being statistically significant, such as heat anomalies being potentially associated with a decreased number of suicide deaths among the younger age group (0–29 years old) and increased deaths among the 30–54 age group.

## Discussion

Our study found that in the period 2000–2019, Australia has been impacted by climate change-induced high heat anomalies substantially larger than previous experience compared with the average from 1950. We identified that out of the suicides recorded from 2000 to 2019, 0.5% were attributable to climate change-induced anomalous heat conditions. This relationship was statistically significant among men aged 55+ years, with 264 suicide death counts (95% CI 257 to 271, p value 0.03). Seasonality emerged as a significant but independent factor of climate change, with suicide risks increasing during spring and summer, aligning with historical sociological findings.^20^ The seasonal increase in suicide occurred regardless of climate change heat anomalies in our fully adjusted model.

The analysis highlights that adults and older adults are susceptible to the effects of temperature anomalies on increased suicide risks, particularly in the range of heat anomalies between 0°C and 3°C with a plateau beyond this range. This is an important finding for public health under climate change, as it may support the promotion of suicide prevention policies and targeted interventions during extreme heat conditions. These include establishing support networks, enhancing access to mental health services and implementing community-based programmes designed to help cope with heat stress.

Another important trend identified in this study indicates that heat anomalies were associated with decreased relative risk among younger people (0–29 years old) despite not presenting statistical significance (p value 0.06 for males and 0.9 for females). The substantial trend of decreased risk of suicide for males aged 0–29 years is surprising and requires further investigation using alternative data sources and methodology, potentially including individual-level factors.

These relationships hold similarities with other studies in other contexts, yet important differences in methods and results are identified. In countries with colder weather, such as Finland, a study using Poisson regression models with data from 1974 to 2010 found that lower temperatures were associated with lower suicide rates among men, with less consistent results for women.^21^ The study authors suggest that thermal stress, particularly during spring with its significant temperature fluctuations, may exacerbate conditions such as depression and increase the risk of suicide.^21^

Similar to our findings, using data from the USA and Mexico, Burke *et al* identified the relationship between rising temperatures and suicide rates.^22^ For each 1°C rise in monthly average temperature, suicide rates were found to increase by 0.7% in the USA and 2.1% in Mexico. It is reported that the effect persists over time and across hotter and cooler regions, suggesting limited historical adaptation to rising temperatures. Despite similarities, our study presents a more nuanced relationship across different age groups. Nevertheless, our study design is based on the population level and does not consider individual factors.^22 23^

Notwithstanding differences, it is essential to highlight alignment with a series of studies in understanding the impact of rising temperatures on well-being, which may worsen due to climate change and associated damages to natural and human systems.^10^ Using a model similar to ours, Kim *et al* analysed the association between daily environmental temperature and suicide deaths in South Korea, taking into account sex, age and education level.^24^ The study identified a relationship between suicide and increased temperatures, especially among males and older adults, which may pose additional risks in a potentially warmer future in a country with suicide rates almost double compared with Australia.^25 26^

The results are consistent with nationwide studies in Taiwan^27^ and Japan,^28^ as well as a multicity study across Japan, South Korea and Taiwan, which suggests that a higher ambient temperature was associated with an increase in suicide in 12 of the 15 cities studied.^29^ Studies from other countries such as France,^30^ England and Wales^31^ show similar results, though England and Wales did not observe the spring or summer peak in suicide rates that our study and others have identified.

In Australia, evidence about the relationship between suicide mortality and temperature is nascent. A study identified the interplay between temperature and unemployment rates on suicide, with higher incidence among individuals identified as Aboriginal and Torres Strait Islanders.^18^ However, despite controlling for age, that study does not present risks associated with different age groups, which was found to be valuable in our context. In addition, we focused on temperature anomalies between the current period and a historical baseline so that variations in heat were identified based on a climate change basis. This aimed to focus on identifying the impacts of climate change on suicide, while others have used the 1°C of increased mean temperature as a metric, which does not consider the expected mean monthly temperature for a specific month of the year for comparison purposes.

An ongoing study in the Perth metropolitan area, building on insights from critical heat studies, demonstrates how extreme heat has detrimental impacts on individuals’ well-being, particularly among high-risk populations. The inability to cope with high temperatures inside and outside of homes, or in their absence, exacerbates mental health issues such as trauma, stress and anxiety among already severely disadvantaged individuals and groups and/or aggravates people’s struggles amid the housing and cost-of-living crises and antipoor policies, all of which are likely to increase the risks of self-harm and suicide attempts.^32^,^35^

Our approach associates suicides with long-term temperature change, including the potential risks associated with increasing temperature anomalies between 2000 and 2019 compared with the period since 1950. In recent years, as demonstrated in our analysis and other studies, climate change is altering the frequency and intensity of temperature anomalies.^36^ This phenomenon should be better explored to improve our understanding of the role of environmental factors in well-being.

This study is subject to several limitations. We used non-linear modelling that identified patterns where suicide rates are higher during periods with temperature anomalies, but this does not imply that temperature anomalies cause an increase in suicide rates. Instead, it points to the complex interplay of various factors, with temperature anomalies being one of the elements associated with fluctuations in suicide rates. In addition, there were some limitations in data collection. First, suicide data was aggregated monthly at the GCCSA level due to privacy and confidentiality considerations of the data custodians. Intricacies and differences within population dynamics within smaller regions or communities are not captured. This lack of additional granularity is explained by the sensitivity associated with suicide data, in which individual data privacy needs to be balanced with utility.^37^ A second limitation is that temperature data was aggregated at the GCCSA level. GCCSAs with large areas characterise Australia, and mean temperatures may not reflect microclimatic variations. In addition, we have not incorporated other climatological variables, such as humidity, which may influence suicide deaths. However, these potential limitations are attenuated by a substantial proportion of Australia’s population being concentrated in large urban areas along the East Coast experiencing similar concurrent variations in humidity and temperature. This implies that there will be correlated experiences of humidity across different subpopulations, as the climatic conditions are relatively uniform within these similar climate zones.

Third, we exclude negative temperature anomalies, as our focus is on the impacts of climate change on suicides associated with high heat. Our study does not investigate the independent question of the potential impacts of unusually lower temperatures. This exclusion is designed to enable our study to capture the relationship between high heat anomalies and suicide. While there is some evidence suggesting that negative temperature anomalies may be less likely to observe increases in suicides and might even be associated with reductions in suicides,^38^ our study is designed to address the public health implications of heat-related stressors specifically. The exclusion of negative anomalies allows us to concentrate on the more prevalent and potentially impactful high-temperature anomalies associated with climate change in Australia.

Fourth, our study is designed as a population-level analysis, focusing on broad trends and associations between temperature anomalies and suicide rates across different demographics and geographic areas. As such, while the study accounts for demographic factors and adjusts for population size, it does not delve into individual-level variables such as socioeconomic status, personal mental health history or area-level parameters such as accessibility to mental health services. This approach aligns with the objective of identifying overarching patterns that can inform public health strategies and interventions at a broader scale. It is essential to recognise that while individual-level factors are critical for a comprehensive understanding of the nuances affecting suicide risk, their inclusion falls outside this study’s scope and methodology. Future research could complement our findings by exploring these individual-level factors in more detail, potentially through case–control or individual cohorts, to provide a granular understanding of the mechanisms underpinning the observed associations.

Finally, while our study provides insight into the Australian context, the findings may not apply to other countries or regions with different climate patterns, socioeconomic conditions or healthcare systems. Despite this, our findings are consistent with the literature supporting the identified relationships elsewhere.

Despite such limitations, our study has several strengths. First, it employs a robust analytical approach to examine non-linear relationships and interactions between climate change-induced heat anomalies and suicide deaths, providing a nuanced understanding of these complex dynamics. Second, our research benefits from a comprehensive dataset that includes detailed population, suicide and temperature data across Australia. Finally, our study contributes to a critical and growing area of research by quantifying the extent to which anomalous heat contributes to suicide mortality. By providing evidence of the measurable impact of climate change on well-being, our research underscores the urgency of integrating climate adaptation strategies into public health planning and suicide prevention efforts.

In conclusion, we found that heat anomalies in Australia during the study period were between 0.48°C and 1.48°C (and between 0.02°C and 2.2°C across GCCSAs) hotter than the historical period due to global warming. These monthly anomalies were associated with increased suicide deaths in some age/sex subgroups. Furthermore, our findings highlight age and sex as important variables, emphasising the necessity for targeted climate change adaptation measures across diverse and often differentially disadvantaged at-risk populations, including layers of intersecting inequalities across society. By understanding multidimensional inequalities and vulnerabilities that drive uneven risk and impacts, policymakers and public health professionals can develop more effective strategies to protect those least able to undertake adaptive and protective actions and reduce risks at the intersection of climatic hazards, exposure and susceptibility. Understanding undesirable and often preventable impacts is imperative to ensure all populations receive support in adapting and protecting themselves and their livelihoods in times of increasingly severe and frequent extreme climatic events.

## Supplementary material

10.1136/bmjment-2024-301131 online supplemental file 1

## Data Availability

Data are available on reasonable request.
