# Supplementary material for: Suicide deaths associated with climate change-induced heat anomalies in Australia: a time series regression analysis
Source: BMJ Ment Health. 2024 Aug 9;27(1):e301131. doi: 10.1136/bmjment-2024-301131 (PMC11409306; doi:10.1136/bmjment-2024-301131)
Supplement: online supplemental file 1 [file bmjment-27-1-s001.pdf]

Supplementary material for

Suicide Deaths Associated with Climate Change-Induced Heat Anomalies in Australia: A Time-Series Regression Analysis

Lucas Hertzog*[1,2,3], Fiona Charlson[4], Petra Tschakert[5], Geoffrey G. Morgan[3,6,7,8], Richard Norman[1,3], Gavin Pereira[1,2,9], Ivan C. Hanigan[1,2,3,8]

[1] Curtin School of Population Health, Faculty of Health Sciences, Curtin University, WA 6102, Australia.
[2] WHO Collaborating Centre for Climate Change and Health Impact Assessment, WA 6102, Australia.
[3] Healthy Environments and Lives (HEAL) National Research Network, Australia.
[4] Queensland Centre of Mental Health Research and School of Public Health, University of Queensland
[5] School of Media, Creative Arts and Social Inquiry, Curtin University, WA 6102, Australia.
[6] School of Public Health, Faculty of Medicine and Health, University of Sydney, Camperdown, NSW 2006, Australia.
[7] University Centre for Rural Health, Faculty of Medicine and Health, University of Sydney, Lismore, NSW 2480, Australia.
[8] Centre for Safe Air, NHMRC CRE
[9] enAble Institute, Faculty of Health Sciences, Curtin University, WA 6102, Australia.

*Correspondence:
lucas.hertzog@curtin.edu.au
400.233, Curtin University, Kent St, Bentley WA 6102

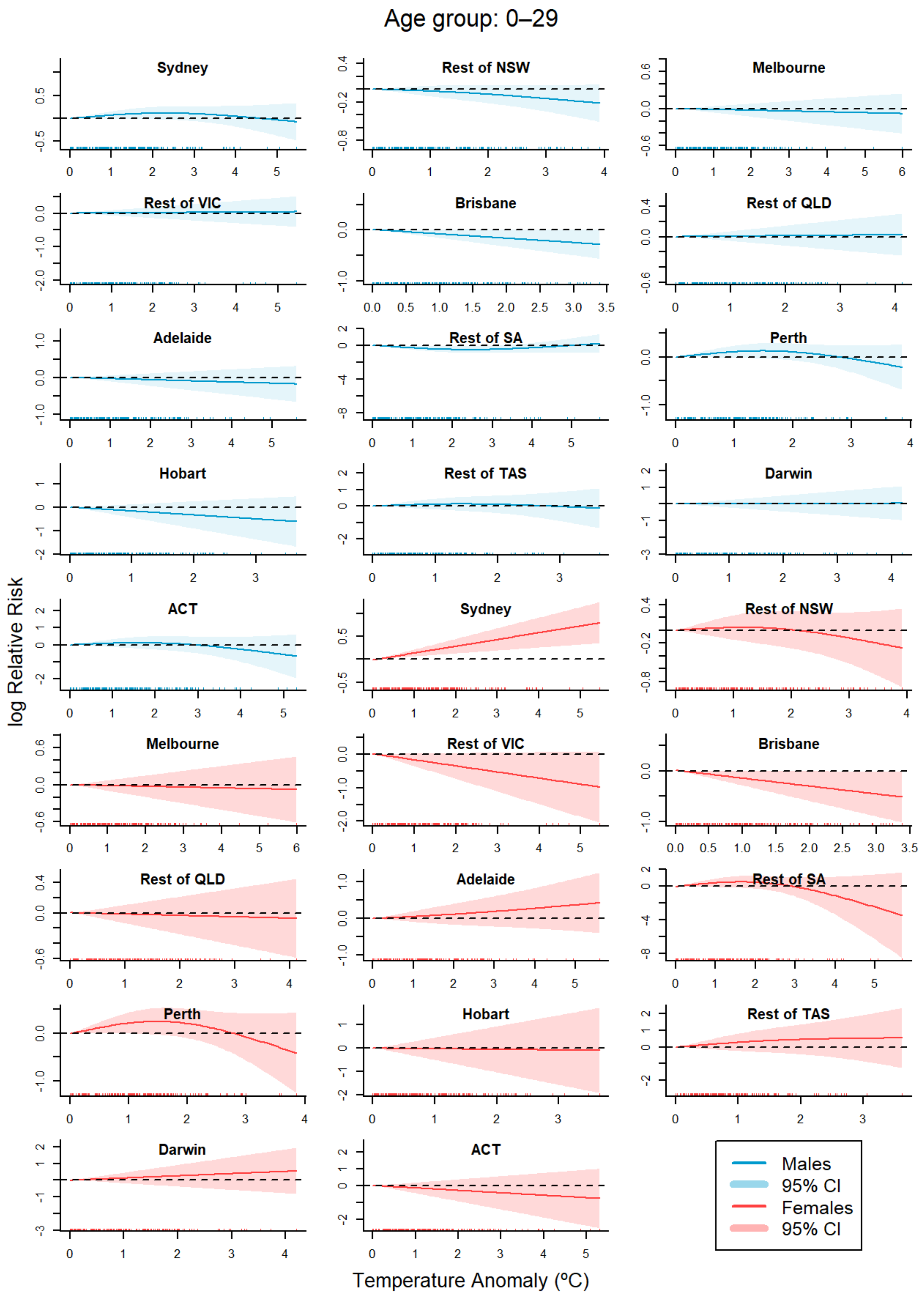

Figure S 1 Association between suicide risk and heat anomaly for the age group from 0 to 29 years, disaggregated by sex and GCCSAs.

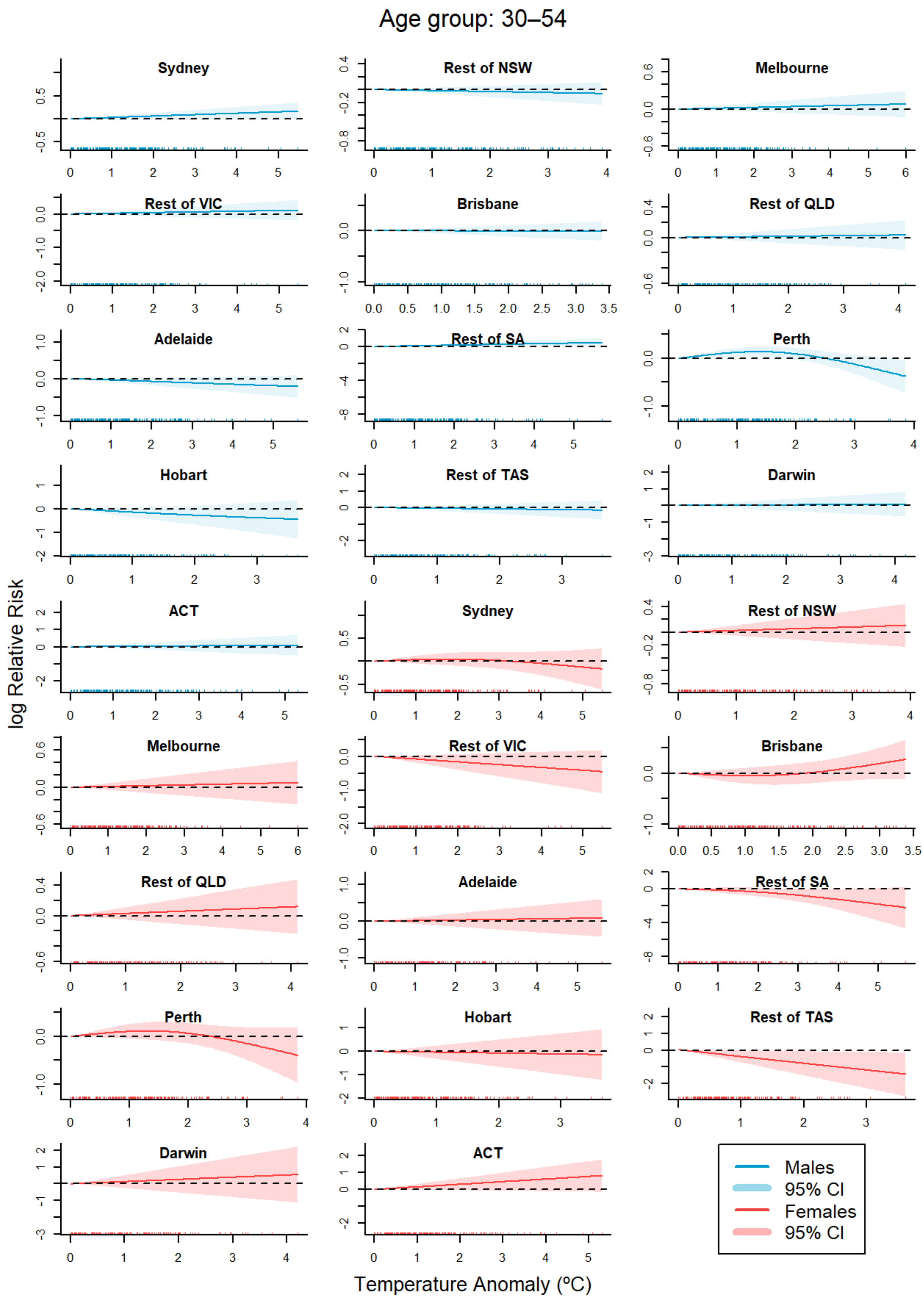

Figure S 2 Association between suicide risk and heat anomaly for the age group from 30 to 54 years, disaggregated by sex and GCCSAs.

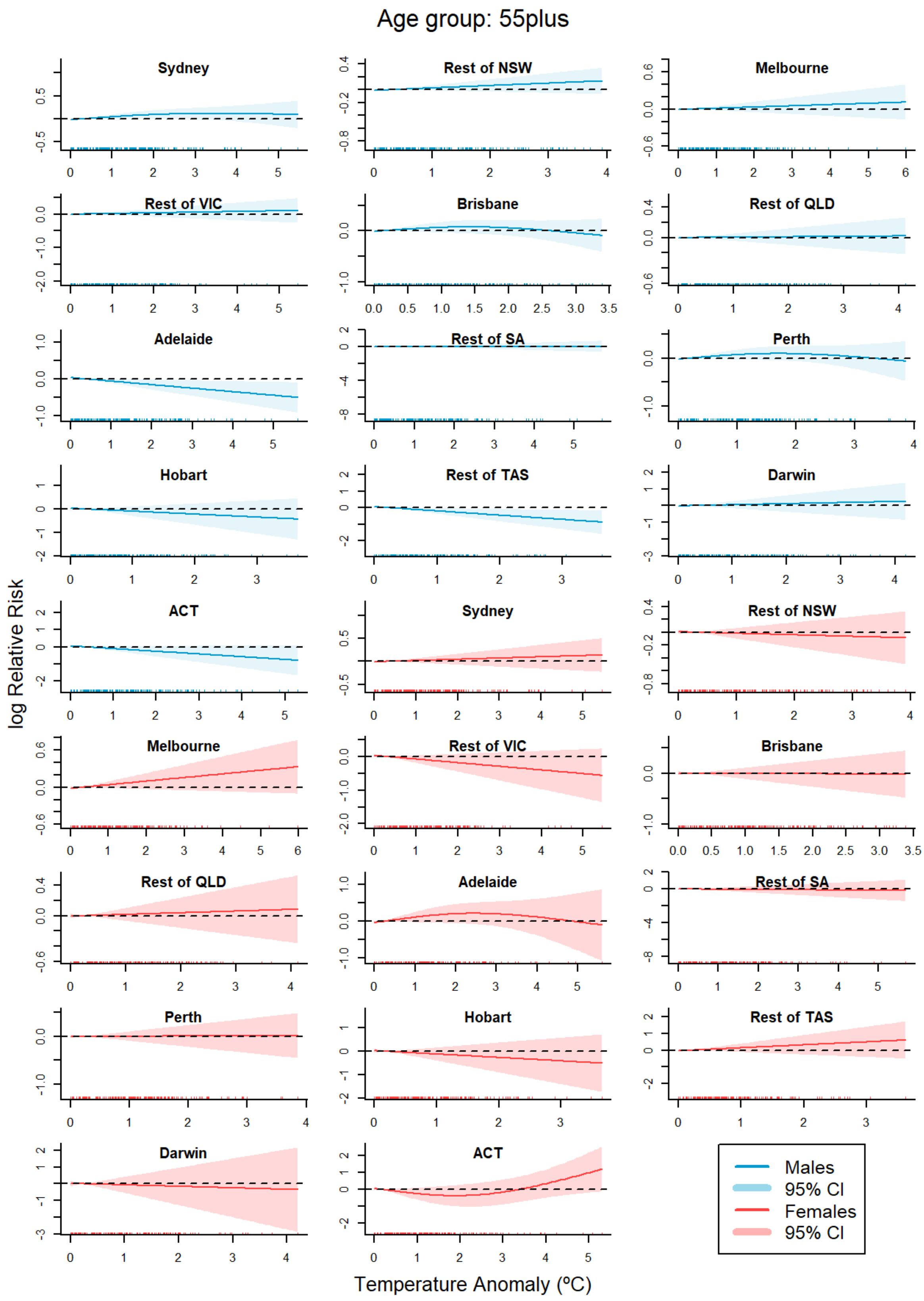

Figure S 3 Association between suicide risk and heat anomaly for the age group with more than 55 years, disaggregated by sex and GCCSAs.
